# Development of Multimorbidity Indexes Based on Common Mental Health Conditions

**DOI:** 10.3389/ijph.2025.1607952

**Published:** 2025-02-12

**Authors:** Junko Kose, Emmanuelle Kesse-Guyot, Pauline Duquenne, Serge Hercberg, Pilar Galan, Mathilde Touvier, Valentina A. Andreeva, Léopold K. Fezeu

**Affiliations:** ^1^ Sorbonne Paris Nord University, Institut national de la santé et de la recherche médicale (INSERM)/Institut national de recherche pour l’agriculture, l’alimentation et l’environnement (INRAE)/Conservatoire national des arts et métiers (CNAM), Center for Research in Epidemiology and Statistics (CRESS), Nutritional Epidemiology Research Team (EREN), Bobigny, France; ^2^ Department of Public Health, Assistance publique – Hôpitaux de Paris (AP-HP) Paris Seine-Saint-Denis Hospital System, Bobigny, France

**Keywords:** mental disorder comorbidity, mental health, multimorbidity index, epidemiological research tool, public health

## Abstract

**Objectives:**

Numerous multimorbidity indexes exist, focused primarily or solely on somatic conditions. We developed mental multimorbidity indexes as epidemiological tools.

**Methods:**

Participants in the French NutriNet-Santé cohort (73.5% women; mean age = 59.5 ± 13.7 years; index development N = 20,000; index comparison N = 7,259) completed self-report questionnaires (2020–2022) regarding depressive symptoms, anxiety, eating disorders, insomnia, alcohol use disorders, cognitive difficulties, and the WHO Disability Assessment Schedule 2.0 (WHODAS 2.0). Using established cutoffs, participants were split into 2 groups for each condition. Tweedie regression analyses were performed with the 6 mental health conditions as exposures and the WHODAS 2.0 score as the outcome. Performance (C-index) and calibration of the indexes were compared with a simple count.

**Results:**

A general and a sex-specific mental multimorbidity indexes were developed; both were significantly associated with the disability score. The new indexes had slightly better predictive performance than simple counts of mental disorders.

**Conclusion:**

We developed mental multimorbidity indexes as epidemiological research tools. Future prospective studies could investigate their predictive potential regarding outcomes such as medication use, healthcare utilization, and quality of life.

## Introduction

From a public health perspective, mental health receives less attention than physical health, despite marked reductions in quality of life (QOL) and life expectancy among individuals with mental illness [1]. The COVID-19 pandemic further worsened the burden of mental disorders [2], which moreover have a high potential for multimorbidity [3] (i.e., coexistence of ≥2 distinct chronic conditions in the same individual). The World Health Organization (WHO) Mental Health Surveys reported a median hazard ratio of 12.1 for onset of a second mental health disorder following an initial diagnosis [4]. The combined morbidity burden of two mental disorders appears to be greater than the sum of the burden estimates for each disorder; multimorbidity is generally associated with increased symptom severity and with a poorer prognosis [3]. The representative U.S. National Comorbidity Survey was among the first to report that high severity occurred in 9.6%, 25.5%, and 49.9% of those with 1, 2, and ≥3 mental disorders, respectively [5]. A latent psychopathological factor, termed the p-factor, has been suggested to help explain mental multimorbidity, given evidence for activation of the same brain regions (e.g., amygdala, hippocampus) in people suffering from different mental illnesses, common genetic expressions, and pathogenesis [6]. In addition, shared socio-demographic and lifestyle risk factors, such as low socio-economic status, unemployment, living in an urban area, low social support, smoking, and unhealthy eating habits, are well documented [7–9]. Thus, there is a compelling need to advance mental multimorbidity research to develop targeted public health interventions.

Multimorbidity assessment is typically based on a disease count or, less frequently, on weighted indexes [10, 11] which account for the number and impact of each condition, providing a better quantification of multimorbidity burden than simple counts [12]. Existing indexes mainly reflect physical conditions and are often age- and sex-standardized [12]. While advancing age is one of the most important risk factors for physical morbidity and multimorbidity [13], that does not seem to be the case with mental illness and multimorbidity [9]. In a systematic review of multimorbidity including 566 studies, <50% included a mental disorder, which was usually major depression and/or dementia [10]. That review identified only 6 studies exclusively focused on mental illness (e.g., major depressive disorder, dysthymia, bipolar disorder, hypomania, panic disorder, various phobias, anxiety disorders, obsessive-compulsive disorder, eating disorders, personality disorders, conduct disorders, attention-deficit hyperactivity disorder, substance abuse/dependence, neurasthenia, cognitive impairment); the inclusion of mental health conditions depended mainly on sample characteristics (e.g., prevalent conditions in outpatients, elderly) and data availability [14–19]. All of these studies relied on mental disorder counts. One study included in that review investigated physical and mental health conditions (depression, anxiety, alcohol abuse, post-traumatic stress disorder, bipolar disorder, schizophrenia) separately and developed a simple count-based Mental Comorbidity Index applicable to war veterans [20]. Indexes measuring somatic and emotional symptoms as adjuncts to clinical diagnosis [21] or as screening tools for dementia [22], have long existed. To our knowledge, no mental multimorbidity index considering common mental health conditions in the general population has been developed.

In turn, commonly used response variables for multimorbidity index development include mortality, primary care consultations, hospitalizations, healthcare expenditures and QOL [12]. No research has addressed the impact of mental multimorbidity on overall disability burden. Our objective was to develop a mental multimorbidity index, assessed against the WHO Disability Assessment Schedule 2.0 (WHODAS 2.0) [23] using data from a French cohort implemented in the general population (described below).

## Methods

### The NutriNet-Santé Cohort

NutriNet-Santé is an ongoing general population prospective cohort initiated in France in 2009 (https://etude-nutrinet-sante.fr/). Its design and specific aims are detailed elsewhere [24]. In short, recurrent multimedia calls target adults who can follow an online research protocol in French. NutriNet-Santé was approved by the Institutional Review Board of the French Institute for Health and Medical Research and by the National Commission on Informatics and Liberty. Eligible volunteers provide informed consent prior to enrollment.

At inclusion and yearly thereafter, participants complete questionnaires regarding socio-demographics and lifestyle, anthropometrics, physical activity and sedentary behavior, dietary intake (every 6 months), and health status. Additional nutrition- or health-related questionnaires are sent on a monthly basis.

### Data Collection

#### Exposure Variables (Mental Health Status)

Mental health conditions meeting the following criteria that reflect public health relevance were identified: relatively high prevalence in the general population, potential for prevention (especially via dietary and lifestyle interventions), treatment or delay of progression, and relatively high risk of premature all-cause mortality. We thus selected depressive disorders, anxiety disorders, chronic insomnia, eating disorders, alcohol use disorders (AUD), and subjective cognitive difficulties.

##### Depressive Symptoms

Depressive symptoms were assessed in 2021 with the 20-item Center for Epidemiologic Studies Depression Scale (CES-D) [25]. The CES-D evaluates depressive symptoms over the previous week. Each item is scored on a 4-point Likert scale ranging from 0 to 3 points [25]. The total score ranges from 0 to 60 points; a score ≥16 identified presence of depressive symptoms. This was the most common cutoff when screening for depression in the general population in various countries [26].

##### Trait Anxiety

In 2020, we administered the trait anxiety subscale of the State-Trait Anxiety Inventory form Y (STAI-T) evaluating general anxiety proneness [27]. The STAI-T consists of 20 items scored on a 4-point Likert scale ranging from 1 to 4 points. The total score ranges from 20 to 80 points [27]. Trait anxiety measured by the STAI-T was reported to be correlated with generalized anxiety disorder [28]. Following prior research, and given the lack of an established cutoff, we used a score >40 for high trait anxiety [29, 30].

##### Chronic Insomnia

Chronic insomnia was assessed using the International Classification of Sleep Disorders – 3rd Edition (ICSD-3) [31] and the Diagnostic and Statistical Manual – 5th Edition (DSM-5) [32] criteria. These items were integrated in the same battery as the STAI-T. Participants experiencing ≥1 sleep problem (difficulties falling asleep, frequent nighttime wakening, waking up too early, unsatisfactory sleep) ≥3 nights/week over the past ≥3 months, and having ≥1 negative repercussion of such problems in daily life were considered as having chronic insomnia.

##### Eating Disorders

Eating disorders were screened with the 5-item Sick-Control-One-Fat-Food (SCOFF) questionnaire [33, 34] which was integrated in the same battery as the STAI-T. Each item is a Yes/No question; ≥2 positive responses indicate a strong likelihood of having an eating disorder (any type) [33, 34].

##### Alcohol Use Disorders (AUD)

During 2021-2022, we administered the 10-item Alcohol Use Disorders Identification Test (AUDIT) which is designed to detect harmful alcohol consumption and alcohol dependence [35]. It contains 3 items measuring frequency, amount of alcohol consumed, and frequency of heavy drinking, and 5 items measuring drinking behavior over the past year. These 8 items are scored on a 5-point Likert scale ranging from 0 to 4 points. The AUDIT also contains 2 items related to alcohol-related problems, scored as 0, 2 or 4 points respectively. The total AUDIT score ranges from 0 to 40 points; a cutoff ≥8 identifies presence of hazardous and harmful alcohol use [35].

##### Subjective Cognitive Difficulties

The Cognitive Difficulties Scale (CDS) was integrated in the same battery as the CES-D [36]. It contains 37 items assessing attention, concentration, praxis, prospective memory, speech problems, memory for personal names, and temporal orientation. Each item is scored on a 5-point Likert scale ranging from 0 to 4 points; the total score ranges from 0 to 148 points. A cutoff score ≥40 (corresponding to the 3rd quartile of the score distribution in our sample) was used to identify participants with pronounced subjective cognitive difficulties [37].

#### Outcome Variable (Disability)

Disability was assessed during 2021-2022 with the WHODAS 2.0 [23] which is regarded as suitable for assessing health status and disability in diverse populations [38]. It is based on the conceptual framework of the International Classification of Functioning, Disability, and Health and consists of 36 items distributed across 6 domains: understanding and communication; getting around; self-care; getting along with people; life activities; and participation in society. Each item has unique scoring; the total score ranges from 0 (no disability) to 100 (complete disability) [23]. WHODAS 2.0 was modelled as a continuous variable.

Supplementary Figure S1 details the mental health assessments.

#### Socio-Demographic, Health Status, and Lifestyle Covariates

Using a validated self-reported socio-demographic questionnaire, we assessed age, sex, education (< high school; high school diploma or equivalent; college/undergraduate degree; graduate degree), employment (without professional activity including homemaker, disabled, unemployed, student; manual/blue-collar worker; office work/administrative staff; professional/executive staff; retired), residential area (rural; semi-urban, urban), marital status (living alone; married/cohabiting), presence of children aged <18 years in the household (yes; no) [39]. A validated self-reported anthropometric questionnaire [40] allowed us to calculate Body Mass Index (BMI; kg/m^2^) and to categorize participants as: underweight: <18.5; normal weight: 18.5–24.9; overweight: 25.0–29.9; with obesity: ≥30.0 kg/m^2^. The International Physical Activity Questionnaire (IPAQ) - Short Form assessed sedentariness (minutes/d spent sitting) and physical activity (low, moderate, high) using established criteria [41]. As all of these questionnaires are administered at baseline and annually thereafter, data recorded closest to the WHODAS 2.0 administration were used for this study. Data on lifetime prevalence (yes; no) of major chronic diseases (cancer except basal cell carcinoma; major cardiovascular diseases; diabetes type 1 or type 2) were collected using an annual health status questionnaire. Information on current household financial difficulties (yes; no), self-perceived health status (very good/good; acceptable; poor/very poor), smoking (never, former or current smoker), and perceived lack of affection during childhood (yes; no) was collected at the same time as AUDIT.

### Statistical Analyses

Given the large number of retired participants (49.5%) to whom four WHODAS 2.0 items regarding work and school activities were not relevant, we excluded these items from the total score, as in prior studies [42]. We calculated scores for the remaining 32 items based on the WHODAS 2.0 algorithm: 100 × (total score for each domain)/92 [23]. Participants with invalid or incomplete mental health or disability data were excluded. To avoid potential acquiescence bias, we also excluded n = 56 for CES-D and n = 39 for STAI-T for marking the same response number for all questions even for inversely-scored items. A total of 27,529 participants were available for the analyses. Using SAS Proc Surveyselect and guided by prior multimorbidity research [43], participants were randomly assigned in a 3 to 1 ratio into one of two groups: a development subsample N = 20,000 to develop the indexes and a comparison subsample N = 7,529 to test and compare the performance of the developed indexes (Supplementary Figure S1). Participant characteristics are presented as number (%) from chi^2^ tests (categorical variables) and median (1st and 3rd quartile) from Wilcoxon’s rank sum tests (continuous variables).

For the index development, we first checked the outcome distribution, expected to be right-skewed [23]. Next, we applied Tobit regression and Tweedie regression (logarithm link function and variance power function = 1.4). Both regression models included the 6 mental health conditions as independent variables and the WHODAS 2.0 score as the dependent variable, with adjustment for age and sex. The former is a regression model appropriate for a dependent variable having either left- or right-censoring [44]. The latter is a regression model appropriate for a dependent variable with positive values and exact zeros, and has been previously used to analyze the WHODAS 2.0 score as an outcome [45]. We compared the performance of the two models given the characteristics of the WHODAS 2.0 score distribution (i.e., dependent variable with positive values and exact zeros, right-censoring), to select the one with a better model fit using Akaike’s Information Criterion (AIC; smaller values reflect a better fit) [46]. Next, interactions of each mental health condition with sex and age were tested with a significance level *p* < 0.10, allowing to balance the reduced statistical power of interaction tests [47] and the risk of committing type I error. Afterwards, two weighting schemes used in the multimorbidity literature were compared by fitting Tweedie regressions with the developed index as the exposure and WHODAS 2.0 total score as the outcome. The first weighting scheme was the beta coefficient (β) multiplied by 10 for each mental health condition [48] and the second was the β divided by the β for age modelled in 5-year increments [49]. Model fit was compared using AIC and the coefficient of determination (R^2^) [50]. Finally, mental multimorbidity indexes calculated with two different rounding systems for the weights (integer and one decimal place) were compared using AIC and R^2^. All model fit comparisons were conducted in the development subsample. The score of the developed index represents the sum of the weights attributed to each mental health condition for each individual.

Next, Tweedie regression models were fitted in the comparison subsample to test whether the developed indexes were associated with disability. Model 1 was unadjusted; Model 2 was adjusted for age and sex; Model 3 was additionally adjusted for confounders selected using a directed acyclic graph (BMI, education, employment, residential area, children in household, lack of affection during childhood, current household financial situation, self-perceived health status, smoking, physical activity, sedentariness, and lifetime prevalence of major chronic disease). Missing covariate values were imputed using the Multiple Imputation by Chained Equations method (20 imputed data sets) [51]. Model fit parameters and predictive performance of the indexes were compared against a simple count of mental health conditions using AIC, R^2^, and calibration plots, respectively [52]. Finally, we assessed discrimination of the indexes (ability to discriminate among individuals with and without disability) by dichotomizing the WHODAS 2.0 score (disability defined as ≥90th percentile in the full sample [53, 54] = 23.9 points) and using Harrell’s concordance index (C-index) corresponding to the area under the receiver operating curve for binary outcomes [52].

All tests were two-sided and *p* < 0.05 reflected statistical significance. The variance power function of the Tweedie regression was determined using the Tweedie package of R version 2.3.5. SAS^®^ version 9.4 (SAS Institute, Inc., Cary NC, United States) was used for all other statistical analyses.

## Results

### Sample Description

NutriNet-Santé participants included in this study were more likely to be male, older, married or cohabiting, to have lower education, higher physical activity, depressive symptoms, anxiety, subjective cognitive difficulties, and lifetime prevalence of chronic conditions compared to their excluded counterparts. Likewise, they were less likely to be retired, to live in urban areas, to have financial difficulties, children in the household, eating disorders, chronic insomnia, or AUD than those excluded from the analyses (data not tabulated; all *p* < 0.01).

Descriptive characteristics and mental health status in the full sample and in each subsample are presented in Tables 1, 2, respectively. Frequency distributions of mental condition pairs are presented in Supplementary Table S1. In the full sample (N = 27,259), mean age was 59.5 ± 13.7 years, 73.5% of the participants were women, and the median WHODAS 2.0 score was 5.4 (Interquartile Range 1.1–13.0). Overall, 58.1% of the participants had ≥1 mental health condition, with the most prevalent pair being anxiety and depressive symptoms; 15.5% and 17.6% of the participants had 2 and ≥3 mental health conditions, respectively. No significant differences regarding participant profiles were observed between the development and comparison subsamples.

**TABLE 1:** Description of participant characteristics (N = 27,259; NutriNet-Santé Study, 2020–2022; France).

	Full sample		Index development subsample		Index comparison subsample		*p*-value^a^
	N = 27,259		n = 20,000		n = 7,259		
Sex							
Women	20,039	(73.5)	14,669	(73.4)	5,370	(74.0)	0.30
Men	7,220	(26.5)	5331	(26.6)	1,889	(26.0)	
Age, years, median (Q1; Q3)	62.0	(50.0; 70.0)	62.0	(50.0; 70.0)	62.0	(50.0; 70.0)	0.68
Age category							
18–39 years	2,875	(10.6)	2,112	(10.6)	763	(10.5)	0.93
40–59 years	9,189	(33.7)	6,729	(33.7)	2,460	(33.9)	
≥60 years	15,195	(55.7)	11,159	(55.8)	4,036	(55.6)	
Educational level^b^							
Less than high school	3,641	(13.4)	2,656	(13.4)	985	(13.6)	0.43
High school diploma or equivalent	4,318	(15.9)	3,138	(15.8)	1,180	(16.3)	
College, undergraduate degree	8,526	(31.5)	6,303	(31.7)	2,223	(30.8)	
Graduate degree	10,616	(39.2)	7,783	(39.2)	2,833	(39.2)	
Employment status^c^							
Without professional activity^d^	1,716	(6.3)	1,213	(6.1)	503	(7.0)	0.05
Manual/blue collar	3,656	(13.4)	2,668	(13.4)	988	(13.6)	
Office work/administrative staff	2,432	(8.9)	1,788	(9.0)	644	(8.9)	
Professional/executive staff	5,933	(21.8)	4,411	(22.0)	1,522	(21.0)	
Retired	13,466	(49.5)	9,882	(49.5)	3,584	(49.5)	
Residential area^e^							
Rural	6,039	(22.5)	4,421	(22.5)	1,618	(22.6)	0.74
Urban or semi-urban	20,802	(77.5)	15,273	(77.6)	5,529	(77.4)	
Self-perceived financial difficulties							
No	23,195	(85.1)	17,064	(85.3)	6,131	(84.5)	0.08
Yes	4,064	(14.9)	2,936	(14.7)	1,128	(15.5)	
Self-perceived health status							
Poor	1,343	(4.9)	960	(4.8)	383	(5.3)	0.24
Acceptable	7,495	(27.5)	5,490	(27.5)	2005	(27.6)	
Good or very good	18,421	(67.6)	13,550	(67.6)	4,871	(67.1)	
Marital status^f^							
Living alone (single, divorced, widowed)	7,542	(27.7)	5,539	(27.8)	2,003	(27.6)	0.87
Married/cohabiting	19,666	(72.3)	14,424	(72.3)	5,242	(72.4)	
Children aged <18 years in household^g^							
No	22,079	(81.2)	16,222	(81.3)	5,857	(80.8)	0.41
Yes	5,128	(18.8)	3,739	(18.7)	1,389	(19.2)	
Self-perceived lack of affection in childhood							
No	23,811	(87.4)	17,478	(87.4)	6,333	(87.2)	0.75
Yes	3,448	(12.7)	2,522	(12.6)	926	(12.8)	
Physical activity level^h^							
Low	4,878	(17.9)	3,572	(17.9)	1,306	(18.0)	0.12
Moderate	10,539	(38.7)	7,669	(38.4)	2,870	(39.6)	
High	11,836	(43.4)	8,755	(43.8)	3,081	(42.5)	
Sedentariness (minutes spent sitting/d), median (Q1; Q3)^i^	360.0	(240.0; 480.0)	360.0	(240.0; 480.0)	360.0	(240.0; 480.0)	0.58
Body Mass Index (BMI; kg/m^2^), median (Q1; Q3)^j^	23.5	(21.2; 26.5)	23.5	(21.2; 26.4)	23.5	(21.2; 26.7)	0.12
BMI category^j^							
Underweight (<18.5)	1,319	(4.9)	960	(4.8)	359	(5.0)	0.11
Normal weight (18.5–24.9)	16,011	(58.9)	11,830	(59.3)	4,181	(57.7)	
Overweight (25.0–29.9)	6,994	(25.7)	5,095	(25.5)	1,899	(26.2)	
Obesity (≥30.0)	2,881	(10.6)	2,075	(10.4)	806	(11.1)	
Smoking status							
Never smoker	17,756	(65.1)	13,018	(65.1)	4,738	(65.3)	0.96
Former smoker	7,490	(27.5)	5,502	(27.5)	1,988	(27.4)	
Current smoker	2,013	(7.4)	1,480	(7.4)	533	(7.3)	
Alcohol use, g ethanol/d, median (Q1; Q3)^k^	2.9	(0; 8.6)	2.9	(0; 8.6)	3.1	(0; 8.6)	0.38
Lifetime prevalence of major chronic diseases^l^							
No	22,150	(81.3)	16,256	(81.3)	5,894	(81.2)	0.87
Yes	5,109	(18.7)	3,744	(18.7)	1,365	(18.8)	
Disability score (WHODAS 2.0), median (Q1; Q3)	5.4	(1.1; 13.0)	5.4	(1.1; 13.0)	5.4	(1.1; 13.0)	0.45

Values refer to number (%) except when noted otherwise.

Values are rounded off to one decimal place except for *p*-values.

^a^ Values are obtained from Chi2 or Wilcoxon’s rank sum tests, as appropriate.

^b^ Educational level contains 158 missing values.

^c^ Employment status contains 56 missing values.

^d^ Without professional activity includes homemakers, disabled, unemployed, and students.

^e^ Residential area contains 418 missing values.

^f^ Marital status contains 51 missing values.

^g^ Children in household contains 52 missing values.

^h^ Physical activity level was assessed with the International Physical Activity Questionnaire and contains 6 missing values.

^i^ Sedentariness contains 7 missing values.

^j^ BMI, contains 54 missing values.

^k^ Alcohol use contains 54 missing values.

^l^ Major chronic diseases: cancer (except basal cell carcinoma), major cardiovascular diseases, and diabetes mellitus type 1 or type 2.

Missing covariate values were imputed prior to the index validation analysis.

**TABLE 2:** Mental health status of participants (N = 27,259; NutriNet-Santé Study, 2020–2022; France).

	Full sample		Index development subsample		Index comparison subsample		*p*-value^a^
	N = 27,259		n = 20,000		n = 7,259		
Depressive symptoms^b^							
No	21,278	(78.0)	15,590	(78.0)	5,688	(78.4)	0.47
Yes	5,981	(21.9)	4,410	(22.1)	1,571	(21.6)	
Anxiety^c^							
Low	18,433	(67.6)	13,524	(67.6)	4,909	(67.6)	0.99
High	8,826	(32.4)	6,476	(32.3)	2,350	(32.4)	
Eating disorders (any type)^d^							
No	24,761	(90.8)	18,169	(90.9)	6,592	(90.8)	0.93
Yes	2,498	(9.2)	1,831	(9.2)	667	(9.2)	
Chronic insomnia^e^							
No	21,416	(78.6)	15,714	(78.6)	5,702	(78.6)	0.97
Yes	5,843	(21.4)	4,286	(21.4)	1,557	(21.5)	
Alcohol use disorders^f^							
No	25,420	(93.3)	18,665	(93.3)	6,755	(93.1)	0.44
Yes	1,839	(6.7)	1,335	(6.7)	504	(6.9)	
Subjective cognitive difficulties^g^							
No	20,178	(74.0)	14,798	(74.0)	5,380	(74.1)	0.84
Yes	7,081	(26.0)	5,202	(26.0)	1,879	(25.9)	
Number of mental health conditions							
0	11,418	(41.9)	8,364	(41.8)	3,054	(42.1)	0.98
1	6,842	(25.1)	5,044	(25.2)	1,798	(24.8)	
2	4,229	(15.5)	3,090	(15.5)	1,139	(15.7)	
3	2,817	(10.3)	2,063	(10.3)	754	(10.4)	
4	1,489	(5.5)	1,099	(5.5)	390	(5.4)	
5	423	(1.6)	309	(1.6)	114	(1.6)	
6	41	(0.2)	31	(0.2)	10	(0.1)	

Values refer to number (%) except when noted otherwise.

Values are rounded off to one decimal place except for *p*-values (two decimal places).

^a^ Values are obtained from Chi2 or Wilcoxon’s rank sum tests, as appropriate.

^b^ Depressive symptoms were assessed with the CES-D scale (cut-off score = 16).

^c^ Anxiety was assessed with STAI-T form Y (cut-off score = 40).

^d^ Eating disorders were assessed with SCOFF (≥2 positive responses = likely eating disorder).

^e^ Chronic insomnia was assessed with a questionnaire based on ICSD-3 and DSM-5 criteria.

^f^ Alcohol use disorders were assessed with the AUDIT scale (cut-off score = 8).

^g^ Subjective cognitive difficulties were assessed with the CDS scale (cut-off score = 40 corresponding to 3rd quartile).

### Index Development

As the Tweedie regression model presented a better fit to the observed data than did the Tobit regression model (AIC = 124,344 for Tweedie; 130,859 for Tobit), the former was retained for subsequent analyses. A significant interaction with sex was observed for AUD (*p* < 0.05) and cognitive difficulties (*p* < 0.07). Thus, we fit separate Tweedie models to develop a general mental multimorbidity index and a sex-specific index.

All mental health conditions were associated with the disability score except for AUD in women (Table 3). The indexes were calculated with two weighting schemes (β*10 and β/β of age for 5-year increments) and with two rounding systems (integer and one decimal place). Those with β*10 (AIC 124,560.9 for β*10 vs. 124,561.1 for β/β of age for 5-year increments) and one decimal place (AIC 124,560.5 for one decimal place vs. 124,574.6 for integer) seemed to provide the best fit especially for the sex-specific index. The final weights attributed to each mental health condition in each index are presented in Table 3. Absolute values of the beta coefficients were generally higher in men than in women, except for depressive symptoms. In both sexes, the highest absolute weight was obtained for subjective cognitive difficulties (6.2 for men, 6.4 for women). The lowest absolute weights were observed for AUD in men (weight = 1.6) and for eating disorders in women (weight = 2.1). The value distribution of the general index (score range: 0–20.5) and the sex-specific index (score range: 0–22.4) is presented in Supplementary Figure S2.

**TABLE 3:** Regression (beta) coefficients and assigned weights for each mental health condition in the full sample and according to sex (index development subsample N = 20,000; NutriNet-Santé Study, 2020–2022; France).

	General index				Sex-specific index							
	n = 20,000				Men (n = 5,331)				Women (n = 14,669)			
	Β^a^	SE^a^	*p*-value^a^	Weight	β^a^	SE^a^	*p*-value^a^	Weight	β^a^	SE^a^	*p*-value^a^	Weight
Depressive simptoms^b^	0.635	0.017	<0.0001	6.4	0.618	0.036	<0.0001	6.2	0.644	0.020	<0.0001	6.4
Anxiety^c^	0.267	0.017	<0.0001	2.7	0.312	0.035	<0.0001	3.1	0.256	0.019	<0.0001	2.6
Eating disorders^d^	0.224	0.022	<0.0001	2.2	0.305	0.051	<0.0001	3.1	0.205	0.024	<0.0001	2.1
Chorionic insomnia^e^	0.219	0.016	<0.0001	2.2	0.225	0.035	<0.0001	2.3	0.219	0.018	<0.0001	2.2
Alcohol use disorders^f^	0.085	0.026	<0.002	0.9	0.157	0.039	<0.0001	1.6	*0.051*	0.034	*0.14*	*0.0*
Subjective cognitive difficulties^g^	0.611	0.015	<0.0001	6.1	0.606	0.028	<0.0001	6.1	0.597	0.020	<0.0001	6.0

Values are rounded off to three decimal places except for *p*-values.

^a^ Values are obtained from Tweedie regression with WHODAS, 2.0 score as dependent variable (adjustment for age and sex for general index; adjustment for age for sex-specific index).

^b^ Depressive symptoms were assessed with the CES-D scale (cut-off score = 16).

^c^ Anxiety was assessed with STAI-T form Y (cut-off score = 40).

^d^ Eating disorders were assessed with SCOFF (≥2 positive responses = likely eating disorders).

^e^ Chronic insomnia was assessed with a questionnaire based on ICSD-3 and DSM-5 criteria.

^f^ Alcohol use disorders were assessed with the AUDIT scale (cut-off score = 8).

^g^ Subjective cognitive difficulties were assessed with the CDS scale (cut-off score = 40).

β, Beta coefficient; SE, Standard error.

### Index Comparison

The general and the sex-specific indexes were positively associated with disability in the crude, age- and sex-adjusted, and the fully-adjusted Tweedie regression models (Table 4).

**TABLE 4:** Associations between mental multimorbidity indexes and disability score (index comparison subsample N = 7,259; NutriNet-Santé cohort, 2020–2022; France).

		β	95% CI	p-value^a^
General mental morbidity index	Crude model	0.107	0.097–0.104	<0.0001
	Model adjusted for age and sex	0.102	0.098–0.106	<0.0001
	Full model^b^	0.079	0.075–0.082	<0.0001
Sex-specific mental morbidity index	Crude model	0.107	0.097–0.104	<0.0001
	Model adjusted for age and sex	0.102	0.098–0.106	<0.0001
	Full model^b^	0.079	0.075–0.082	<0.0001

Values are rounded off to three decimal places except for *p*-values.

^a^ Values are obtained from Tweedie regression models with WHODAS 2.0 score as dependent variable.

^b^ Full model adjusted for age, sex, body mass index, educational level, socio-professional category, area of residence, presence of children in household, lack of affection during childhood, current household financial situation, self-perceived current health status, smoking status, physical activity level, sedentariness, and lifetime prevalence of major chronic disease.

β, Beta coefficient; CI, Confidence interval.

Both indexes showed somewhat lower AIC and higher R^2^ values than those obtained with a simple count of mental disorders (Supplementary Table S2). Absolute values of the discrimination parameters (C-index) were also slightly higher than those of a simple count (Table 5). Calibration plots showed that the developed indexes had slightly better calibration than a simple count of mental disorders (Supplementary Figure S3).

**TABLE 5:** Discrimination of mental multimorbidity indexes and simple count of mental health conditions (index comparison subsample N = 7,259; NutriNet-Santé Study, 2020–2022; France).

	C-index	95% CI
General mental multimorbidity index	0.814	0.797–0.831
Sex-specific mental multimorbidity index	0.813	0.796–0.830
Simple count of mental health conditions	0.791	0.775–0.809

To calculate the C-index the WHODAS 2.0 score was dichotomized using as threshold the 90th percentile in the full sample (disability: ≥23.9 points).

CI, Confidence interval; C-index, Concordance index.

## Discussion

We developed a general and a sex-specific mental multimorbidity indexes based on common mental health conditions as predictors of general disability. Upon replication and confirmation, these indexes could be used as epidemiological research tools. To our knowledge, these indexes are the first such tools exclusively based on prevalent mental health conditions in the general population. The included mental disorders met the following criteria: a relatively high prevalence in the general population, a potential for prevention (especially via dietary and lifestyle interventions), treatment or delay of progression, and a relatively high risk of premature all-cause mortality. In this study, 33% of participants had ≥2 mental health conditions, indicating a high rate of mental multimorbidity. Generally, individuals with different mental health conditions exhibit activation of the same brain regions (e.g., amygdala, hippocampus), common genetic expressions, and shared socio-demographic and lifestyle risk factors [6–9].

The large majority of existing multimorbidity indexes pertain to somatic conditions and are developed for elderly populations or outpatients [12]; mental disorders and general population samples are rare [10, 12]. Also, existing mental multimorbidity measures are usually based on simple counts, not on weighted indexes [10, 14–19]. Only one mental multimorbidity index, including depression, anxiety, alcohol abuse, post-traumatic stress disorder, bipolar disorder, and schizophrenia, was identified [20]. That disease count-based index was validated against QOL, outpatient visits, and mortality in war veteran outpatients [20]. The mental disorders included in mental multimorbidity studies depend mainly on sample characteristics (high prevalence in the elderly, in war veterans) and data availability [14–19]. Prior studies have included not only common and potentially preventable mental disorders, but also genetic, autoimmune and trauma-related disorders [10, 12]. Our indexes were developed in a sample recruited from the general population and using epidemiological tools, considering the number and relative weight (but not severity) of each condition; they could help identify adults at risk and inform prevention efforts. Existing multimorbidity indexes including physical and mental disorders were developed using outcomes such as risk of mortality, hospitalizations, primary care consultations, healthcare expenditures, and QOL [43, 55, 56]. Ours are the first multimorbidity indexes based on general disability.

We developed two mental multimorbidity indexes - a general and a sex-specific index - owing to significant interactions by sex for certain mental disorders. In our sample, AUD were not pertinent among women; in men AUD had a weight of 1.6; AUD prevalence in men and women was 11.2% and 5.1%, respectively. Overall, we observed sex-specific differences in the absolute weights and relative order of importance of the 6 mental disorders. Absolute weights were higher among men than women, except for depressive symptoms. Depressive symptoms and eating disorders had the highest and lowest weights, respectively, among women; subjective cognitive difficulties and AUD had the highest and lowest weights, respectively, among men. Partly owing to sex differences in socialization (e.g., help-seeking, coping, societal expectations), internalizing disorders are generally more common among women whereas externalizing disorders are generally more common among men [57, 58]. It is therefore possible that men’s mental health status might have an increased impact on subjective disability (i.e., WHODAS 2.0 score) which is an external construct.

The multimorbidity literature dealing with index development has relied on expert decisions regarding weights or has derived weights from regression coefficients [43, 55]. Consistent with prior methodological research, we used β coefficients derived from age- and sex-adjusted models as weights for each mental disorder [12, 59]. No other statistical adjustments were made, to develop indexes that are context-independent and therefore more versatile and robust [43]. The indexes were significantly associated with the disability score in the validation subsample following adjustment for many covariates. Model fit parameters and psychometric performance were compared against those obtained from a simple count of mental disorders, which is commonly used in multimorbidity measurement [10]. Model fit indicators of the indexes were somewhat better (lower AIC, higher R^2^) than those of the simple count. The C-index compared the discrimination potential of the indexes and that of a simple count; values <0.7 are considered as indicating poor discrimination whereas values >0.8 reflect excellent discrimination [60]. Our results suggest that the indexes discriminate between individuals with and without disability somewhat better than does a simple count of mental disorders (C-index = 0.81 vs. 0.79). Calibration plots also indicated that the predictive performances of our indexes, especially the sex-specific index, were slightly better than that of a simple count. Moreover, unlike the count, the weighted indexes account for the respective burden associated with each disorder.

### Limitations and Strengths

We obtained mental health data via self-report questionnaires commonly used in epidemiological research, which is consistent with the intended use of these indexes. Yet, despite the fact that the mental health assessment tools have been validated against diagnostic criteria [25, 27, 31–33, 36], they do not reflect clinical diagnoses; this is a limitation of the study. In addition, CES-D, STAI-T and SCOFF do not distinguish among subtypes of depression, anxiety and eating disorders, respectively. For insomnia, we relied on DSM-5 and ICSD-3 criteria. Future confirmatory research could replicate the analysis using different tools for the same mental disorders assessed in representative adult samples. Overall, mental disorders tend to be under-diagnosed in the general population partly due to stigma and perceived discrimination [61]. Mental health assessment in epidemiological studies can help identify individuals with symptoms even without a clinical diagnosis. Next, consistent with the objectives and with prior research, we used the 90th percentile as cutoff of the WHODAS 2.0 score [53, 54, 62] to identify participants with disability. Another limitation stems from the COVID-19 pandemic, which disrupted the way of life and had a deleterious impact of mental health [63, 64], and which coincided with the 2020–2022 questionnaire administration. However, according to a French national survey, average alcohol and tobacco consumption were fairly stable even during the lockdowns [65]. We can speculate that the context might have had a minimal impact on the measures. Finally, our sample was composed predominantly of women and individuals of higher socio-economic status than the respective proportions in the general population [66, 67], which might have led to an underestimation of the impact of mental disorders on disability. Thus, it will be necessary to replicate and confirm the findings using other study populations.

This study has several important strengths. First, we relied on a rich database with available information on a variety of socio-demographic, lifestyle and mental health characteristics. Next, to our knowledge, this is the first mental multimorbidity index development research based on common mental health conditions meeting specific criteria with relevance to public health, and thus applicable in an epidemiological context across age. Moreover, these are the first multimorbidity indexes based on disability, to which mental disorders have a substantial contribution [23]. Disability was measured using WHODAS 2.0 which is commonly used in epidemiology [23]. Assessment of mental disorders relied on validated instruments [25, 27, 31–33, 36] administered in a large and diverse sample of men and women. The prevalence of each mental disorder and that of mental multimorbidity in this study were consistent with those reported in the literature [5, 68–73]. Further, the performance of two statistical approaches (i.e., Tobit and Tweedie regressions) was compared, allowing us to select the one with the better model-fitting performance (smaller AIC value) to develop the indexes. In addition, several weighting schemes used in the multimorbidity literature were tested to identify the one with the best performance [48, 49]. The developed multimorbidity indexes had good discrimination and calibration, and were significantly associated with disability in fully-adjusted models. These indexes could help identify at-risk individuals without a full clinical examination, thus could provide cheaper and earlier screening.

### Conclusion

We developed a general and a sex-specific mental multimorbidity indexes that could help with needs assessment and prevention. These indexes could help identify at-risk individuals without a full clinical examination, thus could provide earlier and cost-efficient screening. Following validation, future research with representative samples could test their predictive potential regarding health status, medication use, primary care consultations, and QOL in longitudinal analyses.

## Data Availability

Researchers at public institutions can submit a project collaboration request that includes information about their institution and a brief description of the proposed project to: collaboration@etude-nutrinet-sante.fr. All requests are reviewed by the steering committee of the NutriNet-Santé study. In case of approval, a signed data access agreement will be requested and additional authorizations from the competent administrative authorities may be needed regarding human participants’ data protection. In accordance with existing regulations, no personally identifiable data will be made available.

## Abbreviations

AIC, Akaike’s Information Criterion; AUD, Alcohol Use Disorders; AUDIT, Alcohol Use Disorders Identification Test; BMI, Body Mass Index; CDS, Cognitive Difficulties Scale; CES-D, Center for Epidemiologic Studies Depression Scale; CI, Confidence Interval; C-index, Concordance Index; DALY, Disability-Adjusted Life Years; ICSD-3, International Classification of Sleep Disorders – 3rd edition; IPAQ, International Physical Activity Questionnaire; DSM-5, Diagnostic and Statistical Manual – 5th edition; QOL, Quality of life; SCOFF, Sick-Control-One-Fat-Food scale; SE, Standard Error; STAI-T, Trait subscale of the State-Trait Anxiety Inventory; WHO, World Health Organization; WHODAS 2.0, WHO Disability Assessment Schedule 2.0.
