# Supplementary material for: Development of Multimorbidity Indexes Based on Common Mental Health Conditions
Source: Int J Public Health. 2025 Feb 12;70:1607952. doi: 10.3389/ijph.2025.1607952 (PMC11859580; doi:10.3389/ijph.2025.1607952)
Supplement: Supplementary file 1 [file DataSheet1.docx]

**Supplementary material**

**Development of multimorbidity indexes based on common mental health conditions**

**Supplementary Table S1**. Frequency distribution of each pair of mental health conditions among individuals with ≥ 2 conditions (n=4,229; NutriNet-Santé Study; 2020-2022; France)

|  | Full sample | | Index development subsample | | Index comparison subsample | |
| --- | --- | --- | --- | --- | --- | --- |
|  | n | % | n | % | n | % |
| Anxiety^1^ + Depressive symptoms^2^ | 883 | 20.9 | 665 | 21.5 | 218 | 19.1 |
| Anxiety + Chronic insomnia^3^ | 806 | 19.1 | 598 | 19.4 | 208 | 18.3 |
| Anxiety + Cognitive difficulties^4^ | 668 | 15.8 | 495 | 16.0 | 173 | 15.2 |
| Depressive symptoms + Cognitive difficulties | 382 | 9.0 | 282 | 9.1 | 100 | 8.8 |
| Chronic insomnia + Cognitive difficulties | 380 | 9.0 | 276 | 8.9 | 104 | 9.1 |
| Anxiety + Eating disorders^5^ | 246 | 5.8 | 181 | 5.9 | 65 | 5.7 |
| Depressive symptoms + Chronic insomnia | 181 | 4.3 | 138 | 4.5 | 43 | 3.8 |
| Alcohol use disorders^6^ + Cognitive difficulties | 180 | 4.3 | 118 | 3.8 | 62 | 5.4 |
| Eating disorders + Cognitive difficulties | 106 | 2.5 | 80 | 2.6 | 26 | 2.3 |
| Anxiety + Alcohol use disorders | 95 | 2.2 | 58 | 1.9 | 37 | 3.2 |
| Chronic insomnia + Alcohol use disorders | 92 | 2.2 | 74 | 2.4 | 18 | 1.6 |
| Eating disorders + Chronic insomnia | 90 | 2.1 | 55 | 1.8 | 35 | 3.1 |
| Depressive symptoms + Eating disorders | 44 | 1.0 | 28 | 0.9 | 16 | 1.4 |
| Depressive symptoms + Alcohol use disorders | 40 | 0.9 | 19 | 0.6 | 21 | 1.8 |
| Eating disorders + Alcohol use disorders | 36 | 0.9 | 23 | 0.7 | 13 | 1.1 |
| Total | 4,229 | 100.0 | 3,090 | 100.0 | 1,139 | 100.0 |
| Values are rounded off to one decimal place.  ^1^ Anxiety was assessed with the trait anxiety subscale of the State-Trait Anxiety Inventory form Y (STAI-T; cut-off score= 40).  ^2^ Depressive symptoms were assessed with the 20-item Center for Epidemiologic Studies Depression Scale (CES-D; cut-off score= 16).  ^3^ Chronic insomnia was assessed with a sleep health questionnaire that included the insomnia criteria of the International Classification of Sleep Disorders – 3^rd^ Edition (ICSD-3) and the Diagnostic and Statistical Manual – 5^th^ edition (DSM-5).  ^4^ Subjective cognitive difficulties were assessed with the Cognitive Difficulties Scale (CDS; cut-off score= 40).  ^5^ Eating disorders were assessed with the 5-item Sick-Control-One-Fat-Food (SCOFF; ≥2 positive responses= likely eating disorder).  ^6^ Alcohol use disorders were assessed with the 10-item Alcohol Use Disorders Identification Test (AUDIT; cut-off score= 8). | | | | | | |

**Supplementary Table S2**. Model fit comparison: general multimorbidity index, sex-specific multimorbidity index, and simple count of mental health conditions in the index comparison subsample (n=7,259; NutriNet-Santé cohort, 2020-2022; France)

|  | General multimorbidty index | Sex-specific multimorbodity index | Simple count of mental conditions |
| --- | --- | --- | --- |
| AIC | 45022.008 | 45021.292 | 45388.864 |
| R^2^ | 0.264 | 0.262 | 0.215 |
| Values are obtained from Tweedie regression with WHODAS 2.0 score as dependent variable.  AIC: Akaike's Information Criterion, R^2^: coefficient of determination | | | |

**N= 53,167** participants with data on depressive symptoms (CES-D) and cognitive difficulties (CDS) (collected in 2021)

**N= 41,228** participants with data on anxiety (STAI-T), eating disorders (SCOFF) and insomnia (collected in 2020)

**N= 34,139** participants with data on disability

(WHODAS 2.0)

(collected in 2022)

**N= 33,895** participants with data on alcohol use disorders (AUDIT)

(collected in 2021-2022)

Exclusion:

**n= 1,034** with invalid/incomplete data

Exclusion:

**n= 657** with invalid/incomplete data

Exclusion:

**n= 440** with invalid/incomplete data

Exclusion:

**n= 622** with invalid/incomplete data

**N= 27,259** participants with complete data on depressive symptoms, anxiety, eating disorders, insomnia, alcohol use disorders, cognitive difficulties, and disability

**Index comparison**

**analysis subsample**

**n= 7,259 participants**

**Index development analysis subsample**

**n= 20,000 participants**

**Supplementary Figure SF1**. Participant selection flowchart (NutriNet-Santé Study, 2020-2022; France)


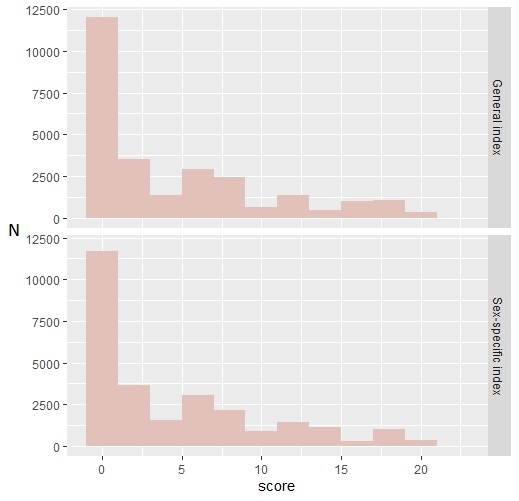


**Supplementary Figure SF2.** Participant distribution according to scores on the general mental multimorbidity index and the sex-specific mental multimorbidity index (N=27,259; NutriNet-Santé Study, 2020-2022; France)

**
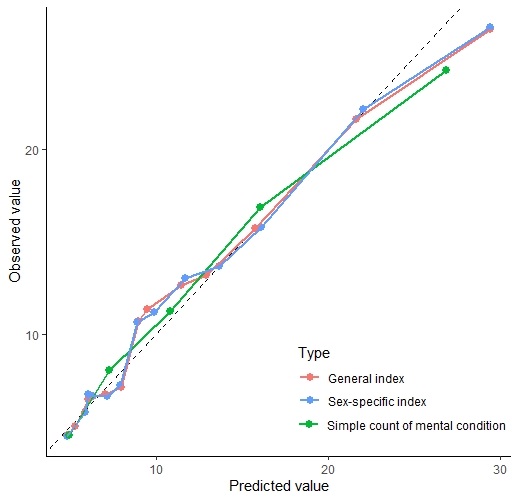
**

**Supplementary Figure SF3**. Calibration plots of the general mental multimorbidity index, the sex-specific mental multimorbidity index and simple counts of mental health conditions in the comparison subsample (n=7,259; NutriNet-Santé Study, 2020-2022; France). Observed values are plotted against predicted values. Dotted line represents perfect calibration.
